# Ubiquitylation and Deubiquitylation in Health and Diseases

**DOI:** 10.3390/biomedicines14051070

**Published:** 2026-05-08

**Authors:** Tadashi Nakagawa

**Affiliations:** 1 Department of Clinical Pharmacology, Faculty of Pharmaceutical Sciences, Sanyo-Onoda City University, Sanyo-Onoda 756-0084, Japan; tnakagaw@rs.socu.ac.jp; 2 Division of Cell Proliferation, United Centers for Advanced Research and Translational Medicine, Graduate School of Medicine, Tohoku University, Sendai 980-8575, Japan

## 1. Introduction

Protein ubiquitylation plays fundamental roles in virtually every aspect of eukaryotic cellular function by regulating the stability and activity of target proteins. Although ubiquitylation has classically been associated with proteasome-mediated protein degradation [1], it is now evident that its functions are far broader, encompassing the recruitment of downstream effectors in signaling pathways [2] and the generation of steric hindrance that disrupts protein–protein interactions [3]. As expected, dysregulated ubiquitylation frequently results in organismal disorders, including cancer and neurodevelopmental disorders (NDDs), thereby providing attractive therapeutic targets [4]. Indeed, proteasome inhibitors are already in clinical use for several malignancies, and a rapidly expanding array of compounds targeting ubiquitin-related pathways are currently under development and evaluation for therapeutic efficacy [5].

Protein ubiquitylation is executed through a canonical three-step enzymatic cascade in which ubiquitin is activated by an E1 enzyme, transferred to an E2 enzyme, and ultimately covalently conjugated to substrate proteins by an E3 ligase. In contrast, deubiquitylating enzymes (DUBs) remove ubiquitin moieties to maintain ubiquitylation homeostasis. The human genome encodes two E1 enzymes, around twenty E2 enzymes, six hundred E3 ligases [6], and one hundred DUBs [7], all of which theoretically represent potential therapeutic targets. Among these, E3 ligases and DUBs are considered particularly attractive owing to their high substrate specificity, underscoring the need for a deeper understanding of E3 ligase and DUB biology to enable the rational development of more effective therapeutics.

**Figure 1:**
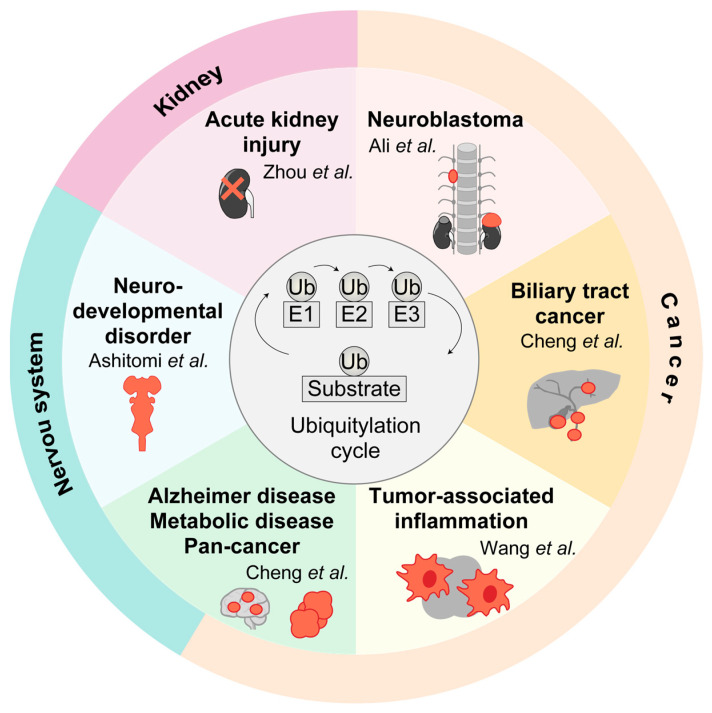
Overview of ubiquitin-dependent regulatory pathways in human diseases highlighted in this Editorial. At the center is a schematic of the ubiquitylation cycle, comprising E1 ubiquitin-activating enzymes, E2 ubiquitin-conjugating enzymes, and E3 ubiquitin ligases, in regulating substrate protein fate. Surrounding disease-specific sectors depict the major pathological contexts addressed in this thematic series. Cancer-related studies include neuroblastoma (Ali et al.) [8], biliary tract cancer (Cheng et al.) [9], and tumor-associated inflammation (Wang et al.) [10], highlighting the contributions of biquitin signaling to tumor progression, immune modulation, and therapy resistance. Additional sectors represent acute kidney injury (Zhou et al.) [11], neurodevelopmental disorders (Ashitomi et al.) [12], and systemic diseases such as Alzheimer’s disease, metabolic disorders, and pan-cancer mechanisms associated with the E2/E3 hybrid enzyme UBE2O (Cheng et al.) [13].

In this context, this Special Issue brings together one original research article and six comprehensive reviews that highlight recent advances in E3 ligase and DUB biology (Figure 1). In this Editorial, I will briefly summarize the articles included in this series to provide an overview of their content and to encourage readers to explore these excellent contributions, which are expected to further stimulate ubiquitin research from both basic and clinical perspectives.

## 2. Ubiquitylation and Deubiquitylation in Cancer

The research article by Ali et al. employs proximity-dependent labeling in neuroblastoma cells to identify novel interaction partners of the deubiquitylating enzyme USP44 [8]. The authors identified 146 proximal proteins and uncovered a previously unrecognized interaction between USP44 and *BRCA2*, a well-established tumor suppressor. Cellular validation revealed that loss of USP44 induces Fanconi anemia-like chromosomal breaks and aberrations, implicating USP44 as a critical regulator of DNA repair. Moreover, high *BRCA2* expression in neuroblastoma was shown to be associated with poor prognosis, closely correlating with enhanced tumor proliferative capacity. Collectively, these findings reveal a previously unappreciated molecular mechanism by which USP44 contributes to cancer development and progression.

Cheng et al. provide a comprehensive review that summarizes the molecular mechanisms by which DUBs contribute to the initiation and progression of biliary tract cancer (BTC) and evaluates their potential as therapeutic targets [9]. It details how individual DUBs (e.g., USP1, USP3, USP7, USP8, USP9X, USP21, and USP22) regulate cancer cell proliferation, metastasis, metabolic reprogramming, and drug resistance. Particular emphasis is placed on oncogenic mechanisms mediated through the stabilization of key proteins such as PARP1 and SIRT1, as well as on the impact of DUBs on mitochondrial dynamics. The review also discusses prospects for personalized medicine using small-molecule inhibitors and PROTAC-based approaches, highlighting the need for novel therapeutic strategies in combination with existing chemotherapies. Overall, this article provides a conceptual framework for the development of next-generation diagnostic and therapeutic approaches to BTC.

The review article written by Wang et al. describes the structural roles and molecular mechanisms by which the deubiquitylating enzyme USP7 contributes to tumor-associated inflammation [10]. USP7 promotes cancer cell proliferation and immune evasion by regulating the stability of key proteins, including the tumor suppressor p53 and the immune-modulatory factor NF-κB. Specifically, it emphasizes that USP7 fosters a tumor-favorable microenvironment through modulation of macrophage polarization and suppression of T-cell activation. Given that USP7 is overexpressed in multiple cancer types and is implicated in resistance to anticancer therapies, it has emerged as a promising therapeutic target. This review also discusses the potential of USP7 inhibition as a future innovative strategy for cancer treatment.

## 3. An E2/E3 Hybrid Enzyme UBE2O in Several Diseases

Cheng et al. also comprehensively describe the structural features of UBE2O, an E2/E3 hybrid enzyme, and its roles in a wide range of human diseases [13]. UBE2O directly regulates protein ubiquitylation and is significantly involved in the progression of cancer, Alzheimer’s disease, and metabolic disorders. In solid tumors and leukemia, UBE2O functions as an oncogenic factor by promoting tumor growth and radioresistance through substrate degradation, whereas in multiple myeloma, it exerts suppressive effects, underscoring its context-dependent roles across disease types. This review further highlights its regulatory functions in skeletal muscle metabolism and neurodegenerative processes. Finally, the authors emphasize the therapeutic potential of targeting UBE2O, including possible interventions using existing agents such as arsenic trioxide, and stress the importance of developing UBE2O-based therapeutic strategies and biomarkers.

## 4. Ubiquitylation in Neurodevelopment

Ashitomi et al. summarize the roles of Cullin–RING E3 ligase (CRL) complexes in brain development and NDDs [12]. They detail how ubiquitin-mediated protein degradation orchestrates all stages of brain development, ranging from neural stem cell proliferation to neuronal migration and synapse formation. Furthermore, they demonstrate—using behavioral analyses in mouse models—that mutations in specific genes such as CUL3 and CUL4B are implicated in the pathogenesis of NDDs. The article concludes that the elucidation of these molecular mechanisms will pave the way for the development of future therapeutic strategies.

## 5. Ubiquitylation and Deubiquitylation in Kidney Disease

The review article by Zhou et al. explores the roles of the ubiquitin and ubiquitin-like modifiers (UBLs) in the pathogenesis of acute kidney injury (AKI) [11]. The authors emphasize that these systems do not operate as independent pathways but instead form intricately coordinated regulatory networks that govern inflammatory responses, cell death, and mitochondrial dysfunction. Specifically, the review details how the E1–E2–E3 enzymatic cascade and DUBs play central roles in cellular survival and repair processes. It further highlights that ubiquitylation-related post-translational modifications, including SUMOylation and neddylation, influence renal cell fate and are implicated in disease progression. The article proposes that targeting these regulatory mechanisms through novel drug development and precision medicine approaches may represent innovative therapeutic strategies for AKI.

## 6. Emerging Technologies for Identifying Substrates

Matsuhisa et al. highlight state-of-the-art technologies for identifying ubiquitylation targets [14]. Because E3 ligases recognize substrates with high specificity and are closely involved in disease pathogenesis, accurate identification of their targets is indispensable for therapeutic research. The authors focus on proximity-labeling approaches using biotin ligases, such as BioID, as powerful methods to capture transient interactions that are difficult to detect with conventional techniques. Their review compares the characteristics of advanced enzymes, including TurboID and AirID, and summarizes how these tools have been applied to systematic substrate discovery. In addition, it discusses their contributions to next-generation drug development strategies, such as PROTACs, and the potential of genome editing to analyze target proteins under physiological conditions. The authors emphasize that these emerging technologies will serve as key drivers for the development of novel therapeutic strategies for cancer and neurodegenerative diseases.

## 7. Concluding Remarks

Collectively, the seven articles highlighted in this Editorial underscore the central role of ubiquitin-dependent regulatory systems in human disease, spanning neurodevelopmental disorders, cancer, inflammation, acute organ injury, and genome maintenance. By covering diverse molecular layers, including E2/E3 ligases, DUBs, UBLs, and emerging proximity proteomics technologies, these studies illustrate how finely tuned ubiquitin signaling governs cell fate decisions under both physiological and pathological conditions. The integration of advanced methodologies, particularly proximity labeling-based proteomics, further highlights a shift toward system-level mapping of ubiquitin networks in native cellular contexts. Together, these works emphasize that ubiquitin signals should be viewed not as isolated enzymatic events but as interconnected regulatory circuits with broad biological and clinical consequences. I anticipate that the concepts and approaches presented in this Special Issue will stimulate further interdisciplinary research and accelerate the development of therapeutic strategies targeting ubiquitin-mediated pathways across a wide spectrum of diseases.

## Data Availability

No new data were created in this study.
